# Evaluation of cancer immunotherapy using mini-tumor chips

**DOI:** 10.7150/thno.71761

**Published:** 2022-05-01

**Authors:** Zheng Ao, Hongwei Cai, Zhuhao Wu, Liya Hu, Xiang Li, Connor Kaurich, Mingxia Gu, Liang Cheng, Xin Lu, Feng Guo

**Affiliations:** 1 Department of Intelligent Systems Engineering, Indiana University, Bloomington, IN 47405, United States; 2 Perinatal Institute, Division of Pulmonary Biology, Cincinnati Children's Hospital Medical Center, Cincinnati, OH 45229, United States; 3 Center for Stem Cell and Organoid Medicine, CuSTOM, Division of Developmental Biology, Cincinnati Children's Hospital Medical Center, Cincinnati, OH 45229, United States; 4 University of Cincinnati School of Medicine, Cincinnati, OH 45229, United States; 5 Department of Pathology and Laboratory Medicine, Indiana University School of Medicine, Indianapolis, IN 46202, United States; 6 Department of Biological Sciences, Boler-Parseghian Center for Rare and Neglected Diseases, Harper Cancer Research Institute, University of Notre Dame, Notre Dame, IN 46556, United States; 7 Indiana University Melvin and Bren Simon Cancer Center, Indianapolis, IN 46202, United States

**Keywords:** Tumor-on-a-chip, tumor microenvironment, cancer immunotherapy, personalized therapy

## Abstract

**Rationale:** Predicting tumor responses to adjuvant therapies can potentially help guide treatment decisions and improve patient survival. Currently, tumor pathology, histology, and molecular profiles are being integrated into personalized profiles to guide therapeutic decisions. However, it remains a grand challenge to evaluate tumor responses to immunotherapy for personalized medicine.

**Methods:** We present a microfluidics-based mini-tumor chip approach to predict tumor responses to cancer immunotherapy in a preclinical model. By uniformly infusing dissociated tumor cells into isolated microfluidic well-arrays, 960 mini-tumors could be uniformly generated on-chip, with each well representing the *ex vivo* tumor niche that preserves the original tumor cell composition and dynamic cell-cell interactions and autocrine/paracrine cytokines.

**Results:** By incorporating time-lapse live-cell imaging, our mini-tumor chip allows the investigation of dynamic immune-tumor interactions as well as their responses to cancer immunotherapy (e.g., anti-PD1 treatment) in parallel within 36 hours. Additionally, by establishing orthotopic breast tumor models with constitutive differential PD-L1 expression levels, we showed that the on-chip interrogation of the primary tumor's responses to anti-PD1 as early as 10 days post tumor inoculation could predict the *in vivo* tumors' responses to anti-PD1 at the endpoint of day 24. We also demonstrated the application of this mini-tumor chip to interrogate on-chip responses of primary tumor cells isolated from primary human breast and renal tumor tissues.

**Conclusions:** Our approach provides a simple, quick-turnaround solution to measure tumor responses to cancer immunotherapy.

## Introduction

Over the past decade, tremendous efforts have been made to develop novel cancer treatments, with over 130 new cancer drugs approved by the United States Food and Drug Administration (FDA) and more worldwide 1. Novel cancer adjuvant therapies have evolved from generic cytotoxic drugs to targeted therapy and immune therapy, requiring more characterization of the primary tumor to define the best treatment scheme for the patient 2-4. Precision medicine taking into consideration of predictive assays to guide treatment was shown to improve progression-free survival (PFS) and overall survival (OS) of cancer patients 5-8. Currently, cancer biomarkers for adjuvant therapy mainly rely on the genetic makeup or gene expression profiles of the primary tumors. Such molecular biomarkers are proven effective in targeted therapy with well-defined molecular targets 9-13. Cancer immunotherapy, in contrast, can be affected not only by the molecular expression of the drug target (e.g., PD1/PD-L1 expression in immune checkpoint inhibition therapy) but also by the tumor mutational burden as well as the complex and dynamic immune components at play. Thus, it remains a grand challenge to predict patients' responses to certain immunotherapy.

*Ex vivo* cultures of a tumor, unlike molecular biomarkers which represent only a single "genetic snapshot" of tumors by the time of resection, could preserve tumor components viably. The viable cells can then be subject to various treatments for drug response prediction treatment selection 14-19. 2D reprogrammed tumor cell cultures or 3D organoids have demonstrated promising potential since they preserve tumor cell genetic makeup as well as a transcriptomic profile with high fidelity 20-26. However, 2D cultures and 3D organoid cultures with a medium favoring tumor cell growth disregard other tumor microenvironment (TME) components, such as stromal cells and immune cells. An alternative is to subject already established organoids to immune cell infiltration, where T cells were added to the organoid culture to mimic peripheral immune cell infiltration and generate tumor-reactive T cells 27, 28. However, the organoid cultures need a prolonged process that always takes more than 7 days. During this process, the original primary tumor residing TME components such as tumor-associated macrophages (TAMs), dendritic cells (DCs), and myeloid-derived suppressor cells (MDSCs) may lose either viability and/or functional phenotypes 29, 30. Additionally, the microfluidic organotypic culture of tumor spheroids and air-liquid interface culture of multicellular tumor spheroids have been explored to successfully preserve TME components 19, 31-35. However, these methods rely on the gentle dissociation of tumors into random-sized multicellular spheroids. The resulting tumor spheroid size and components vary highly from spheroid to spheroid, and each spheroid can contain a very distinct component makeup, making it difficult to evaluate treatment efficacy and compare multiple treatments in parallel *ex vivo*. Additionally, the *ex vivo* culture lacks the compact structure as well as intratumor cytokine concentration that is crucial for TME maintenance. Thus, an assay that could preserve all TME components *en bloc,* while amendable to real-time monitoring of *ex vivo* drug treatment efficacy is highly desirable for cancer disease management and personalized therapy.

Here, we report a mini-tumor chip to consist of massive microwell arrays for assessing tumors' treatment responses in parallel. The primary tumor digested single-cell suspensions can be flowed into this mini-tumor chip and settle down into 960 mini-tumors (or cell clusters) by gravity. Each mini-tumor could be a representative niche of all TME components, allowing for on-chip immune-tumor interaction and preserving local cytokine concentrations from autocrine and paracrine signaling. The mini-tumor chip design allows for real-time monitoring of on-chip treatment efficacy and is compatible with downstream analysis such as flow cytometry and cytokine analysis, with a fast readout within 24 h post tumor resection. Utilizing this chip, we were able to predict tumor responses to immune checkpoint inhibitors (ICI) in animal models as early as 2 weeks post tumor cell inoculation. We also demonstrated on-chip evaluation of the patient primary tumors' response to ICI. To conclude, our mini-tumor chip can serve as an *ex vivo* assay that preserves tumor microenvironment components and their interactions *en bloc* with a fast readout. It can be utilized as a potential predictive assay for ICI therapy and is also extendable for general personalized therapy applications.

## Results and Discussion

**Figure 1:**
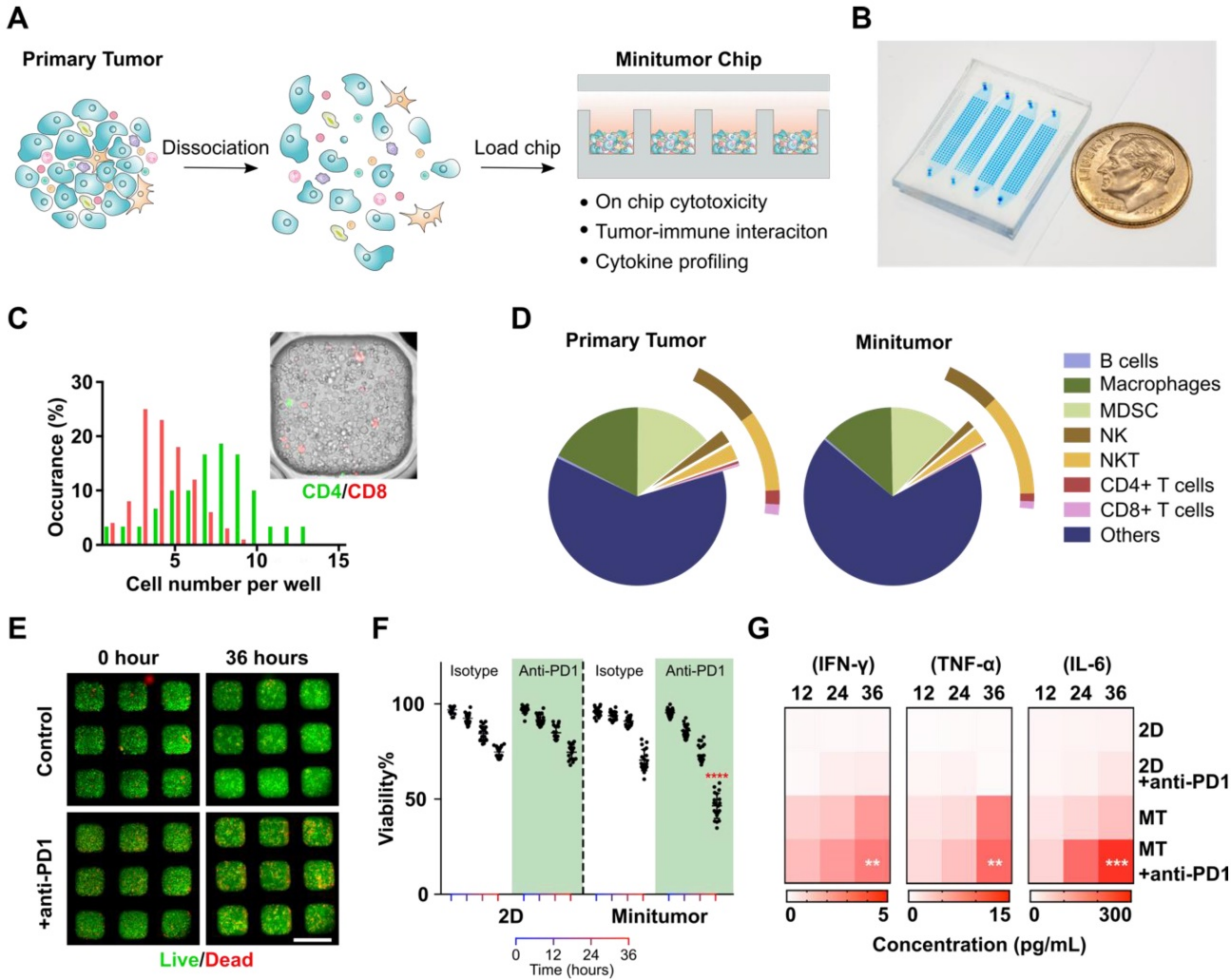
** Work principle of the mini-tumor chip. (A)** The design concept of the mini-tumor chip. **(B)** Image of the mini-tumor chip with 4 parallel injection ports. **(C)** Distribution of CD4+ and CD8+ T cells within mini-tumor wells. **(D)** Cellular makeup comparison between primary tumor and cells loaded into the mini-tumor chip. **(E)** Viability measurement under control and anti-PD1 treated conditions. **(F)** Quantification of on-chip cell viability over time under control and anti-PD1 treatment conditions in 2D cultures and mini-tumor chips. Data points from anti-PD1 treated groups were compared with corresponding controls at the same timepoint by student's t-test (n = 20, ****p < 0.001). **(G)** Cytokine secretion profile (IFN-γ, TNF-α, IL-6) of ex vivo culture under control and anti-PD1 treatment conditions in 2D cultures and mini-tumor chips. Data points from anti-PD1 treated groups were compared with corresponding controls at the same timepoint by student's t-test (n = 3, **p < 0.01, ***p < 0.005). Scale bar: 500 µm.

**Working principle of the mini-tumor chip.** To faithfully recapitulate *in vivo* tumor microenvironment (TME) status, we designed our mini-tumor chip with 3 key features: (1) Massive microwell arrays containing 16 channels with 60 wells per channel to profile 960 mini- aggregations of tumor cell components, each aggregation containing an independent yet evenly distributed and comprehensive representation of all cell components inside the primary tumor. (2) 16 parallel injection ports to allow interrogation of 16 independent treatment conditions per sample. (3) Semi-separate aggregation wells allow efficient cell perfusion, and *in situ* cell-cell interaction while preserving local cytokine gradients (**Figure 1A-B**).

**Validation of mini-tumor chip.** To optimize and validate our mini-tumor chip, we first explored the relationship between infusion cell concentrations and cell number per mini-tumor microwells. We discovered that the cell number per microwell increased proportionally to infusion cell concentration and plateaued around 1809 ± 81 cells per well at an infusion concentration of 12.5 million cells per milliliter (**Figure S1**). The cells form 3D clusters within the 400 µm X 400 µm well with an approximate thickness of 150 µm on the Z-axis. By flow analysis (**Figure S2),** we discovered that in a typical primary tumor (orthotopic EO771 tumor), CD8+ T cells have a frequency of 0.27±0.14% (Supplementary). Assuming a CD8+ T cell frequency of 0.27%, if CD8+ T cells distribution across microwells follows Poisson distribution, our chip guarantees a 99.2% of chance of at least one CD8+ T cell per well. As all other common TME cells make up a larger proportion of the dissociated tumor cells, the probability to guarantee even distribution is higher. To further validate this calculation, we stained CD4+ and CD8+ T cells with pre-conjugated antibodies and enumerated their distribution across microwells, the observed distribution frequency confirmed our calculation (**Figure 1C**). Additionally, we compared the cell composition of the primary tumor components with flowed-out cells from our mini-tumor chip by flow cytometry. The data indicated that cell components on our mini-tumor chip can represent that of the primary tumor (**Figure 1D**).

We next examined on-chip tumor viability in response to anti-PD1 treatment. We quantified cell viability by time-lapse live-dead staining. We confirmed that the cells on-chip can maintain high viability over the initial 24 h on-chip, with a slight viability drop at 36 h. To test whether the 3D microenvironment could better recapitulate the tumor's drug responses, we treated anti-PD1 or isotype controls to the tumor cells loaded onto our mini-tumor chip versus in a 2D culture 96 well plate (see methods). Anti-PD1 treatment efficacy was not observed in 2D cultures (36 h viability isotype control: 74.2 ± 2.8% versus anti-PD1 treatment 74.1 ± 3.9%, p = 0.94). In contrast, tumor cells cultured in mini-tumor chips showed a marked response to anti-PD1, as evidenced by the 36 h viability difference (isotype control: 69.9 ± 5.6% versus anti-PD1 treatment 45.8 ± 6.6%, p < 0.0001) (**Figure 1E-F**). This is likely due to the compact cell aggregation allowing for cell-cell interaction as well as concentrated cytokines. We further measured proinflammatory cytokine levels in supernatant from 2D tumor cell cultures or flow-out from mini-tumor chips at various timepoints. We observed an increase in all cytokine concentrations in mini-tumor chips versus 2D culture. Furthermore, this cytokine difference is further amplified by anti-PD1 treatment (**Figure 1G**).

**Figure 2:**
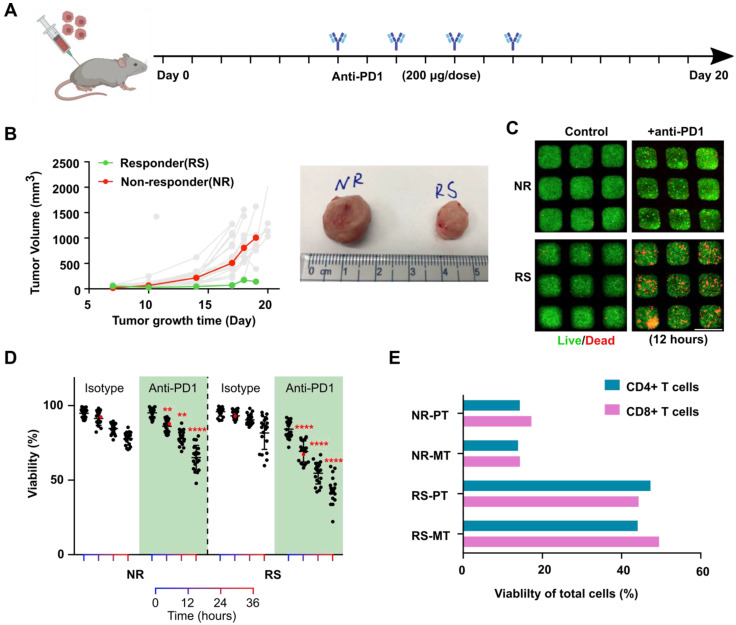
** Profiling of tumor responses to anti-PD1 blockade using the mini-tumor chip. (A)** Treatment scheme of EO771 tumor-bearing mice. **(B)** Orthotopic EO771 tumor growth curve with representative responder (RS) and non-responder (NR) tumor. **(C)** Live/dead staining images of RS and NR tumors with on-chip anti-PD1 treatment for 12 h and quantitative analysis. Scale bar: 500 µm. **(D)** Viability of RS and NR tumors on-chip. Data points from anti-PD1 treated groups were compared with corresponding controls at the same timepoint by student's t-test (n = 20, **p < 0.01, ****p < 0.001), viability data points of representative images shown in Fig 2C were highlighted in red color. **(E)** Flow analysis of CD4+ and CD8+ T cell composition of EO771 dissociated primary tumors and on-chip cell components pre-treatment with both RS and NR tumors.

**Profiling of tumors' response to anti-PD1 treatment.** To further validate our chip and demonstration its application for the evaluation of tumor responses to anti-PD1 treatment. We established an orthotopic EO771 tumor model in syngeneic C57BL6 mice (n = 20) and subject the tumor-bearing animals to a series of anti-PD1 treatments (**Figure 2A**). Intrinsically, orthotopic EO771 tumors display heterogeneous growth and drug responses to anti-PD1 treatment 36. With our xenograft initial cell number and treatment scheme (see methods), we observed that the majority of the EO771 tumors were resistant to anti-PD1 treatment. Occasionally, we observed a few tumors that responded well to the anti-PD1 treatment (**Figure 2B**). We then isolated the orthotopic tumor from an outlier responder, where tumor volume regresses at day 18 (RS, final tumor volume at day 19: 143.6 mm^3^) as well as a typical non-responder, where tumor volume shows continuous growth despite anti-PD1 treatment (NS, final tumor volume at day 19: 1006.6 mm^3^). The dissociated single tumor cells from both tumors were loaded onto our mini-tumor chips and treated with anti-PD1 or isotype controls and visualized the on-chip cell death by live/dead staining (**Figure 2C**). After 12 h of on-chip treatment of anti-PD1, a significant difference between RS and NR on-chip viability can be observed (12 h viability RS: 69.3 ± 7.1% versus NR: 86.2 ± 4.3%, n = 20, p < 0.0001), which further differed as on-chip culture was maintained with longer time (36 h viability RS: 43.3 ± 8.7% versus NR: 65.2 ± 8.2%, n = 20, p < 0.0001) (**Figure 2D**). Eventually, both tumors responded on-chip to anti-PD1 treatment. However, the anti-PD1 treatment for the RS group showed more profound responses as compared to the NR tumor on-chip. Additionally, we analyzed the tumor components makeup of the RS and NR tumors. We found that RS primary tumor has significantly higher makeup of CD4+ and CD8+ T cells as compared with NR tumors (RS-PT: CD4+ T cells 0.47%, CD8+ T cells 0.44% versus NR-PT: CD4+ T cells 0.14%, CD8+ T cells 0.17%). This T cell make up difference is faithfully represented by the Mini-tumor chips (RS-MT: CD4+ T cells 0.44%, CD8+ T cells 0.49% versus NR-MT: CD4+ T cells 0.14%, CD8+ T cells 0.14%) (**Figure 2E**).

**Figure 3:**
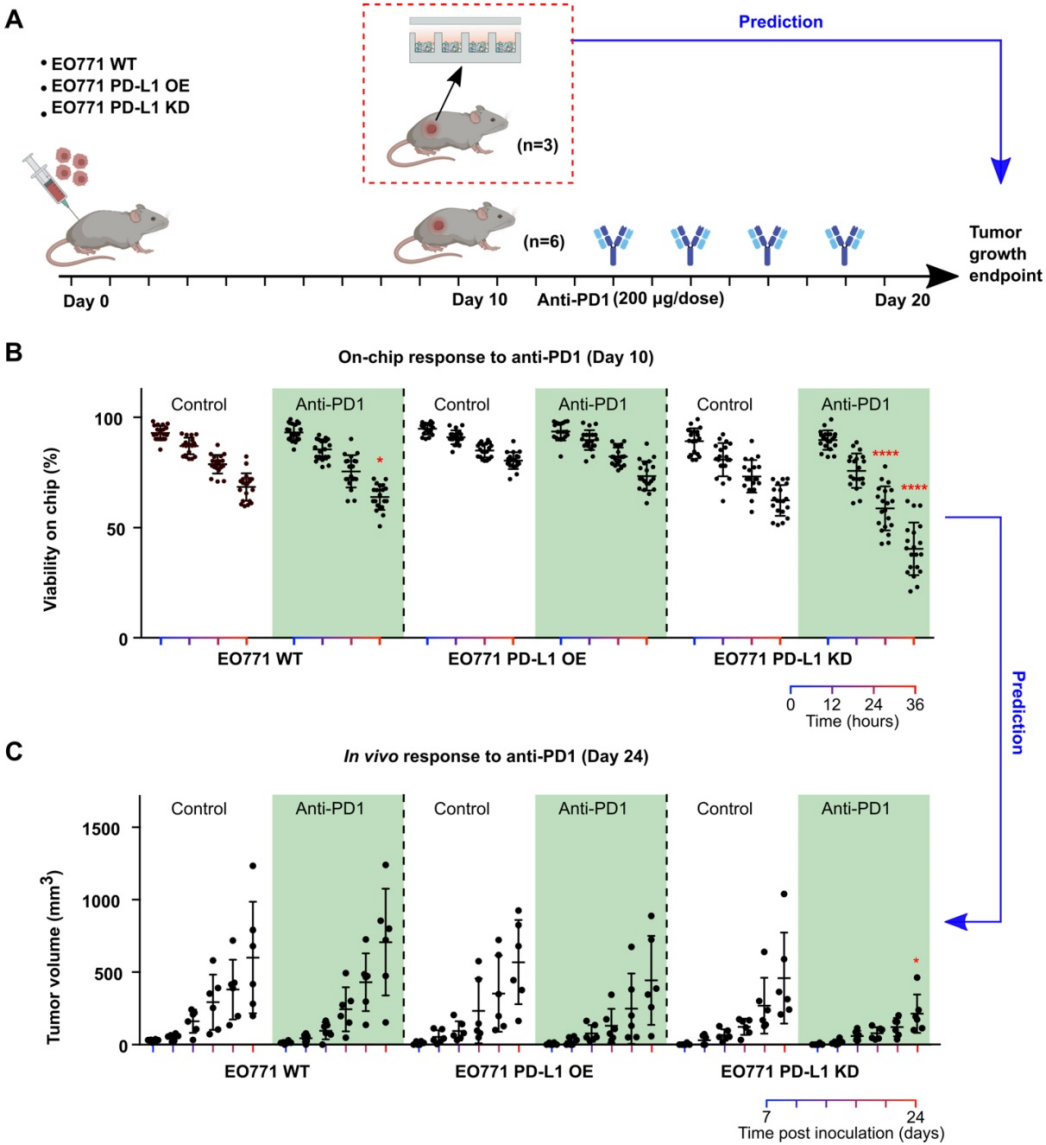
** Prediction of tumors' responses to anti-PD1 treatment using the mini-tumor chip. (A)** Experimental scheme test on-chip prediction of primary tumors' responses to anti-PD1 treatment. **(B)** Dissociated tumor cells on-chip responses to anti-PD1 at day 10, with orthotopic tumors formed by EO771 wild type (WT), PD-L1 overexpression (OE), and PD-L1 knockdown (KD) cells. **(C)** Tumor growth curve of orthotopic EO771 tumors with wild type, PD-L1 overexpression, and knock-down. Data points from anti-PD1 treated groups were compared with corresponding controls at the same timepoint by student's t-test (n = 20, *p < 0.05, ****p < 0.001).

**Prediction of tumors' responses to anti-PD1 treatment.** Patients' tumors can respond to anti-PD1 treatment with high variation due to different tumor composition, T cell infiltration and tumor PD-L1 expression, etc. Thus, it is of great interest to predict patients' responses to anti-PD1 therapies for disease prognosis as well as stratify patients. To mimic the heterogenous responses to anti-PD1 treatment, we engineered EO771 tumor cells to overexpress or under-express PD-L1 by transfecting them with PD-L1 overexpression vector or PD-L1 knockdown shRNA lentiviral vectors. We then xenografted these EO771 cells (wild type: WT; PD-L1 overexpression: OE; PD-L1 knockdown: KD) orthotopically into C57BL6 mice, with 9 mice per group. We then terminated 3 animals per group right after palpable tumors were formed (day 10), and loaded dissociated tumor cells onto Mini-tumor chips to interrogate their responses to anti-PD1 treatment (**Figure 3A**). To our surprise, PD-L1 KD tumors responded the best to anti-PD1 treatment (36 h on-chip viability post-anti-PD1 treatment: WT 63.9 ± 5.8%; OE 73.5 ± 6.6%; KD: 40.4 ± 11.9%, n = 20, One-way ANOVA p < 0.0001) (**Figure 3B**). Upon closer examination, we found that PD-L1 KD tumors hold significantly higher CD3 tumor-infiltrating T cells in comparison with wild-type and PD-L1 OE tumors (**Figure S3**). This could be due to the constitutive knockdown of PD-L1, promoting early T cell infiltration. We then followed up with the tumor growth curve of the rest of the 6 animals in each group. Indeed, PD-L1 KD tumors responded the best to anti-PD1 treatment, consistent with our on-chip prediction (Tumor size at day 24 post-anti-PD1 treatment: WT: 706 ± 367 mm^3^; OE: 443 ± 307 mm^3^; KD: 214 ± 132 mm^3^, n = 6, One-way ANOVA p < 0.05) (**Figure 3C**). This data demonstrated that our mini-tumor chip can predict tumors' response to anti-PD1 treatment as early as 10 days past tumor initiation.

**Figure 4:**
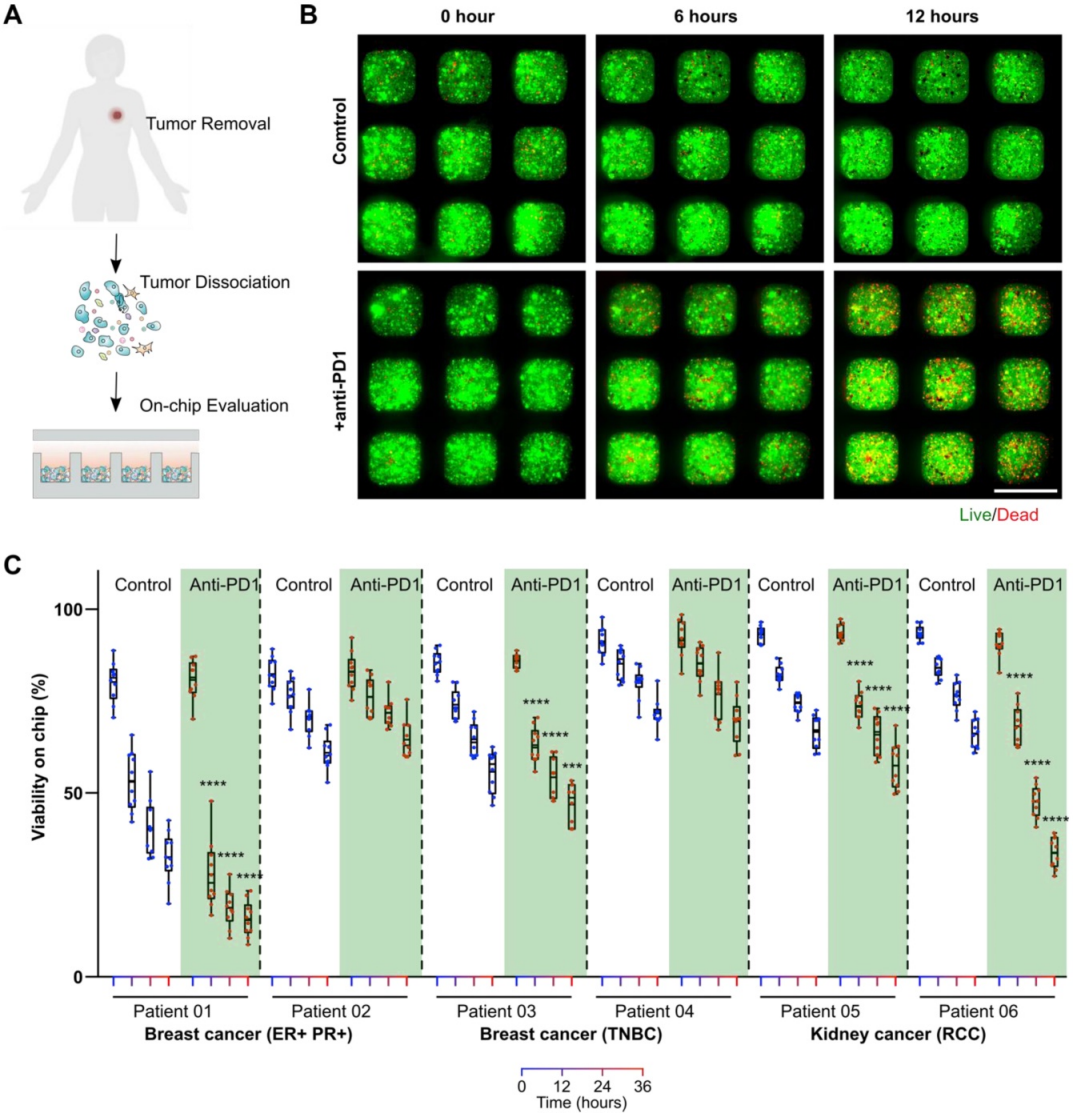
** On-chip monitoring of patient tumor sample responses to anti-PD1 treatment. (A)** Experimental scheme test on-chip prediction of primary tumors' responses to anti-PD1 treatment. **(B)** Dissociated breast tumor cells on-chip responses to anti-PD1. **(C)** Quantification of viability on-chip in the control group and anti-PD1 treated group in estrogen receptor (ER)+, progesterone receptor (PR)+ breast tumor group, triple-negative breast tumor group (TNBC), and renal cell carcinoma (RCC) group. Scale bar: 500 µm. Statistical analysis was performed using Sidak's multiple comparisons test: p-value, ***p < 0.005, ****p < 0.001

**On-chip monitoring of patient primary tumors' responses to anti-PD1 treatment.** To test whether our mini-tumor chip could be utilized for on-chip testing of ICI treatments for clinical samples, we harvested patient primary tumors from patients with estrogen receptor and progesterone receptor-positive (ER+ PR+) breast cancers or triple-negative breast cancers (TNBC) and patients with renal cell carcinomas (RCC). Primary tumors were dissociated into single cells and loaded on a chip. We then treated primary cells on mini-tumor chips with isotype antibody controls or pembrolizumab (human anti-PD1 antibody drug) (**Figure 4A**). On-chip cell viability was monitored for 36 h (**Figure 4B**). We found that primary tumor cells from patients with ER+ PR+ breast cancers, TNBC as well as RCC could respond to anti-PD1 treatment on-chip with various efficiency (**Figure 4C**). This on-chip response highlights our chips' utility to interrogate patient tumors' responses to ICI and the potential for future personalized therapy applications.

## Discussion

Evaluation and prediction of patients' responses to therapy are critical to stratify them for the right treatment and prolong their survival. Personalized therapy based on companion diagnostic tools holds great potential for clinical applications and may be critical for successful treatments in the era of precision medicine. Traditionally, companion diagnostic tests are based on traditional pathology slides or more recently, with molecular profiling such as DNA-sequencing. Such tools, although effective for treatments with well-known molecular targets, could face challenges with novel therapies especially immune therapies, which efficacy is affected by multi-facet, dynamic tumor microenvironment (TME). *Ex vivo* culture or xenografting tumor fragments into animals could serve as an alternative method to profile tumor TME. However, it is time-consuming, labor-intensive, and often has a low success rate, thus limiting its utility as companion diagnostics.

Here, we present a novel microfluidics-based mini-tumor chip. Based on parallel flow units, dissociated tumor components could be aggregated into 960 tumor cell clusters with similar composition as primary tumor whereas allowing local cell-cell interaction and preserving autocrine and paracrine signals. We demonstrated that on-chip responses to anti-PD1 could reflect that of the primary tumors in a preclinical model. Utilizing EO771 syngeneic cell lines with engineered differential immune checkpoint (PD-L1) expression, we demonstrated that the responses observed on-chip predict the responses of *in vivo* tumors with similar PD-L1 expression levels which were assessed two weeks later. This data demonstrated the potential utility of our assay to analyze needle biopsy samples from patients' primary tumors for personalized therapy selection. Finally, we tested primary tumors from breast and renal cell carcinoma patients on-chip. Tumor cells from both tumor types showed responses to anti-PD1 treatment. This further validated our assay's potential for clinical use. One limitation of our current study is that all patient samples analyzed were resected tumors, where treatment of ICI would be less likely following such surgery. A more comprehensive and representative evaluation is required to better validate our current platform by analyzing patient needle biopsy samples on the chip before ICI treatment and correlating on-chip response to ICI to their clinical responses.

Currently, the mini-tumor chip still lacks several features such as tumor matching extracellular matrix, which has been demonstrated to affect tumor growth, drug responses, and immune infiltration 37, 38. Additionally, modeling tumor vasculature and structure on-chip could also facilitate understanding the TME and develop immune-oncology therapies 39, 40. Furthermore, currently, tumor cells on-chip start to lose viability after 36 h, better medium perfusion with the constant flow as well as fine-tuning the on-chip culture medium to include essential cytokines such as interleukin-2 may further enhance on-chip culture time and avoid non-specific cell death.

Overall, our mini-tumor chip demonstrated excellent clinical utility in quickly testing patient primary tumors' responses to anti-PD1 treatment. We envision this test could be widely adopted for personalized therapy. Additionally, our method consists of small units of primary tumors with only ~2,000 cells per aggregate. A typical tumor fragment of 1 milligram could yield ~20,000,000 cells, which could be used to form 20,000 individual mini-tumor units on-chip. This could be adapted to high-throughput testing of novel therapies, especially those targeting tumor microenvironment cells such as cancer-associated fibroblasts, tumor-associated macrophages, dendritic cells, etc.

## Materials and methods

**Mini-tumor chip fabrication.** The mini-tumor chip was designed with drafting software (AutoCAD) and fabricated using our well-developed SU8 lithography and PDMS fabrication method 41. The mini-tumor chip consists of three sample loading inlets (or more depending on the application) and 1,000 wells, each of which has a dimension of 400 μm × 400 μm × 320 μm (length × width × height). Minitumor chips were autoclaved and pre-treated with 70% ethanol to allow easy injection.

**EO771 tumor model establishment.** All *C57BL6* mice were purchased from Envigo. All animal experiments and procedures are approved by Indiana University Bloomington Institutional Animal Care and Use Committee (BIAUC). To establish the orthotopic EO771 model, 500,000 EO771 cells were harvested from culture and resuspended in 50% RPMI-1640/50% matrigel (Corning). The cell mixture is then injected into the mammary fat pad of female *C57BL6* mice 5 weeks old. Anti-mouse-PD1 (Clone RMP1-14) was injected peri-tumor starting 1-week post tumor cell injection and bi-weekly at a dose of 200 µg. Animals were euthanized on day 21 or if the tumor's longest diameter reached 1.3 mm.

**Tumor cell culture and genetic editing.** EO771 tumor cells were purchased from the American Type Culture Collection (ATCC) and maintained in RPMI-1640 supplemented with 10% fetal bovine serum (FBS) and 100 unit/milliliter Penicillin-Streptomycin, in a 37ºC incubator with 5% CO_2_. To establish PD-L1 knock-down EO771 cell line, EO771 cells were transfected with GIPZ Lentiviral shRNA targeting mouse CD274 (Horizon, Clone ID: V2LMM_62208). Single clones of transfected cells were selected by puromycin and validated by flow cytometry. To establish PD-L1 overexpression EO771 cell line, EO771 cells were transfected pUNO1-mCD274 vector expressing mouse PD-L1 (Invivogen #puno1-mcd274). Single clones of transfected cells were selected by blasticidin and validated by flow cytometry.

**Collection and digestion of mouse primary tumors.** Primary tumors were resected from tumor-bearing mice, washed twice with sterile Dulbecco's Phosphate Buffered Saline (DPBS), and subject to tumor digestion using a mouse primary tumor dissociation kit and gentle MACS dissociator (Miltenyi) following manufacturer manuals.

**Collection and digestion of human primary tumors.** All human tumors were collected under a protocol approved by Indiana University Institutional Review Board (IRB # 1907977109). Freshly resected primary tumors were subject to tumor digestion using a human primary tumor dissociation kit and gentle MACS dissociator (Miltenyi) following manufacturer manuals.

**Loading mini-tumor chips with dissociated tumor cells.** Dissociated single cells were stained with live CSFE dye at 5 µM for 15 min and washed twice with 1XDPBS and resuspended in RPMI 1640 medium (Gibco) supplemented with 10% fetal bovine serum (Gibco) and penicillin-streptomycin (100U/mL) (Gibco). Tumor cells were pipetted slowly at a concentration of 12.5 million cells per milliliter onto the mini-tumor chip with 1 μM ethidium homodimer-1 (EthD-1) in 10 μL volume for each injection port (16 parallel injection ports in total per tumor). Mini-tumor chip was then imaged using an inverted fluorescent microscope (Olympus IX-81) inside an incubation chamber (Tokai hit) set at 37 ºC, 5% CO_2_. For 2D controls, 10 μL volume of cells were loaded at the same concentration (12.5 million cells per milliliter) into one well of 96 wells and topped with an additional 40 μL of medium to avoid well from drying. For anti-PD1 treatment, the injection cell medium was supplemented with anti-mouse PD1 (Bio X Cell, Clone RMP1-14) at a concentration of 3.5 µg/mL.

**Flow cytometry analysis.** Dissociated tumor cells or on-chip tumor cells collected were resuspended in 1XDPBS supplemented with 2mM EDTA and 0.5% bovine serum albumin. The single tumor cell suspension was incubated with fluor-conjugated antibody (Supplementary Table) at 4 °C for 30 min and washed twice before being analyzed on a BD LSRII flow cytometer. All analyses were performed with FlowJo v10.

**Cytokine analysis.** 10 µL supernatant was collected from the minitumor chip (10 µL total volume per chip) or 2D control conditions (50 µL total volume per one well in 96 well plates) and brought up to 50 µL volume by adding 40 µL RPMI-1640 medium, diluting the minitumor chip supernatant to the same volume as 2D control supernatant. Cells are pelleted by centrifugation at 500 g at 4 °C for 10 min. Supernatants were then pipetted and shipped to Abcam on dry ice and analyzed by the FirePlex (Biolegend) analysis.

## Supplementary Material

Supplementary figures and table.
