# Supplementary material for: Evaluation of cancer immunotherapy using mini-tumor chips
Source: Theranostics. 2022 May 1;12(8):3628–36. doi: 10.7150/thno.71761 (PMC9131272; doi:10.7150/thno.71761)
Supplement: Supplementary file 1 — Supplementary figures and table. [file thnov12p3628s1.pdf]

Supplementary Information

Evaluation of cancer immunotherapy using mini-tumor chips

Zheng Ao,[1] Hongwei Cai,[1] Zhuhao Wu,[1] Liya Hu,[1] Xiang Li,[1] Connor Kaurich,[1]

Mingxia Gu,[2,3] Liang Cheng,[4,6] Xin Lu,[5,6] and Feng Guo[1,6]*

1. Department of Intelligent Systems Engineering, Indiana University, Bloomington, IN 47405, United States
2. Perinatal Institute, Division of Pulmonary Biology, Cincinnati Children's Hospital Medical Center, Cincinnati, OH 45229, United States
3. Center for Stem Cell and Organoid Medicine, CuSTOM, Division of Developmental Biology, Cincinnati Children's Hospital Medical Center, Cincinnati, OH 45229, United States
4. Department of Pathology and Laboratory Medicine, Indiana University School of Medicine, Indianapolis, IN 46202, United States
5. Department of Biological Sciences, Boler-Parseghian Center for Rare and Neglected Diseases, Harper Cancer Research Institute, University of Notre Dame, Notre Dame, IN 46556, United States
6. Indiana University Melvin and Bren Simon Cancer Center, Indianapolis, IN 46202, United States

*Corresponding email: fengguo@iu.edu

Supplementary Figures

- **Figure S1. Optimization of injection cell concentration into minitumor chip.**
- **Figure S2. Immune cell profiling of cells inside EO771 primary tumor.**
- **Figure S3. TIL quantification of EO771 wild type (WT), PD-L1 over-expression (PD-L1 OE) and PD-L1 knock down (PD-L1 KD) primary tumors.**
- **Figure S4. Evaluation of function of tumor microenvironment (TME) components in minitumor on-chip.**
- **Figure S5. Comparison of tumor components from dissociated primary tumor cells and tumor cells on-chip.**
- **Figure S6. Cytokine analysis of dissociated tumor cells culture ex vivo in 96 well plate (2D) and minitumor chip**

Supplementary Tables

- **Table S1. Antibody used in flow cytometry analysis**

Supplementary Figures

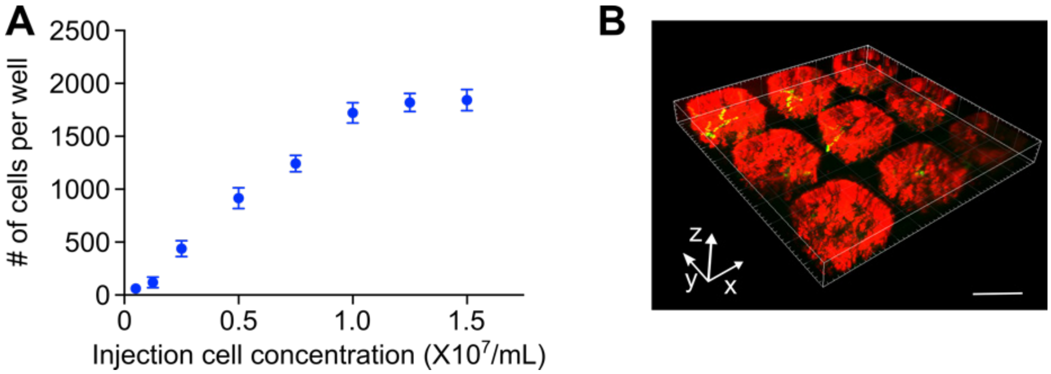

Figure S1. Optimization of injection cell concentration into minitumor chip. (A) Optimization of injection concentration of minitumor chip using EO771 cell line. Various concentrations of EO771 cells were pre-labeled with CFSE and injected into minitumor chip (n=3). Cell numbers for each condition were enumerated using a fluorescent microscope. **(B)** Tumor cells (red) and T cells (green) form 3D cell clusters in mini-tumor chip visualized by confocal microscopy. Scale bar: 200 µm.

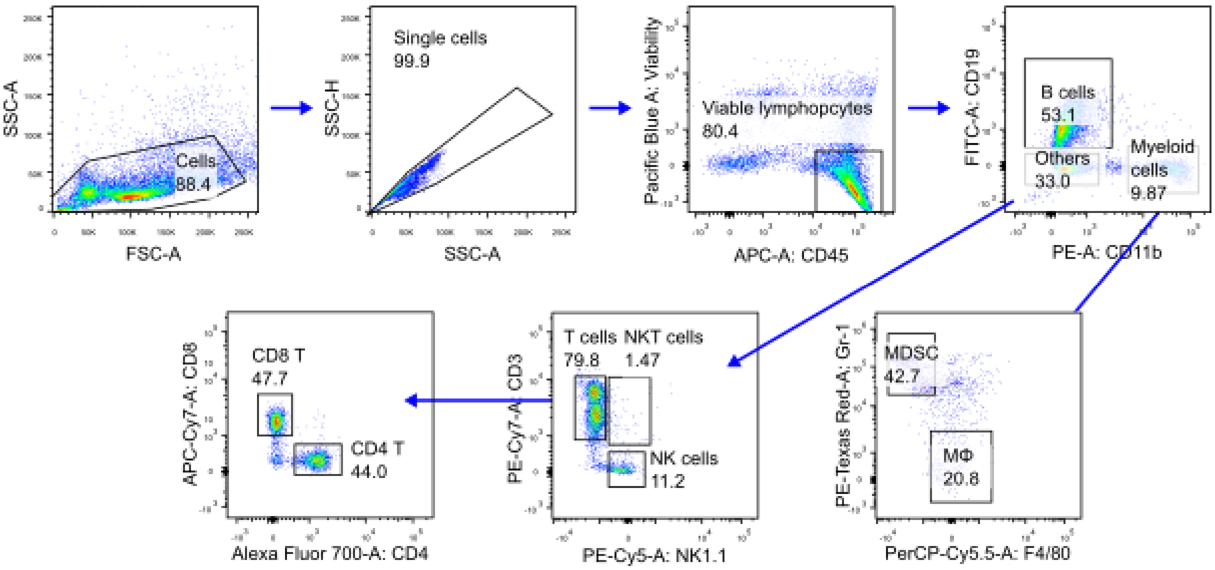

Figure S2. Immune cell profiling of cells inside EO771 primary tumor. Gating strategy used to profile EO771 primary tumor components. EO771 spleen cells were used as staining and gating controls.

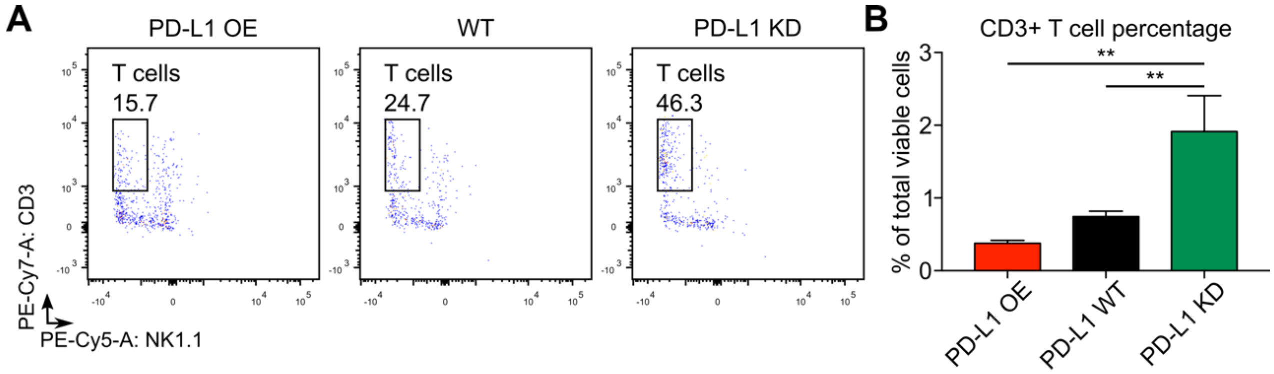

Figure S3. TIL quantification of EO771 wild type (WT), PD-L1 over-expression (PD-L1 OE) and PD-L1 knock down (PD-L1 KD) primary tumors. We quantified CD3+ TIL percentages inside EO771 WT, OE and KD primary tumors (n=3) at day 10 post tumor inoculation. Tumors with PD-L1 knock down showed significantly higher TIL infiltration (One-way ANOVA, post-hoc Tukey's test, p**<0.01).

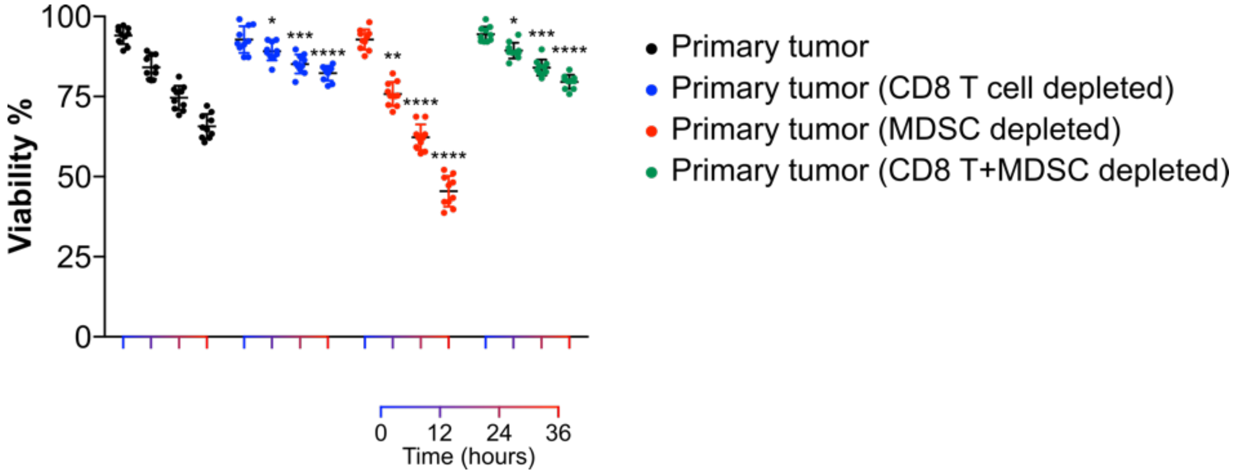

Figure S4. Evaluation of function of tumor microenvironment (TME) components in minitumor on-chip. To evaluate function of TME cells on-chip, we depleted T cells by CD8 magnetic beads (Miltenyi 130-116-478), myeloid derived suppressor cells (MDSC) by Ly6G magnetic beads (Miltenyi 130-094-538) or co-depleted both. Where depletion of CD8 T cells reduced on-chip cell death, depletion of MDSC by Ly6G promoted on-chip cell death, which is abolished by T and MDSC co-depletion. Data points from cell component removed groups were compared with the control (Primary tumor) at the same timepoint by student's t-test (n=20, *p<0.05, ****p<0.001).

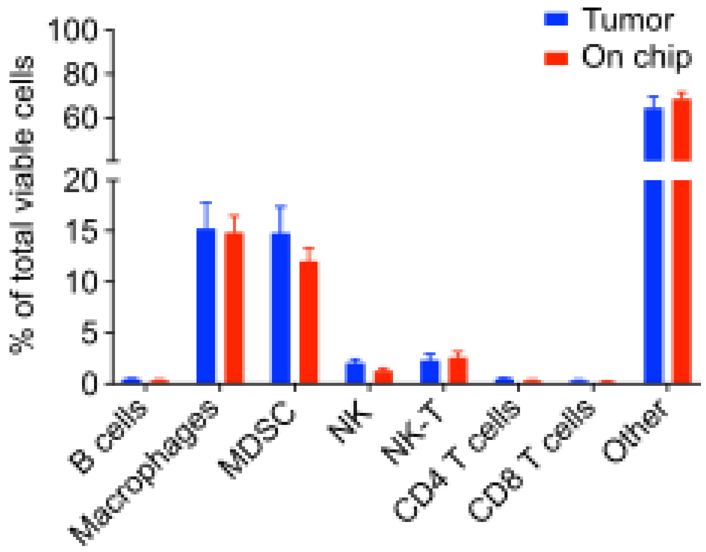

Figure S5. Comparison of tumor components from dissociated primary tumor cells and tumor cells on-chip. Bar graph representing percentage of various cell components in primary tumor and tumor cells on chip as depicted in Fig 1D.

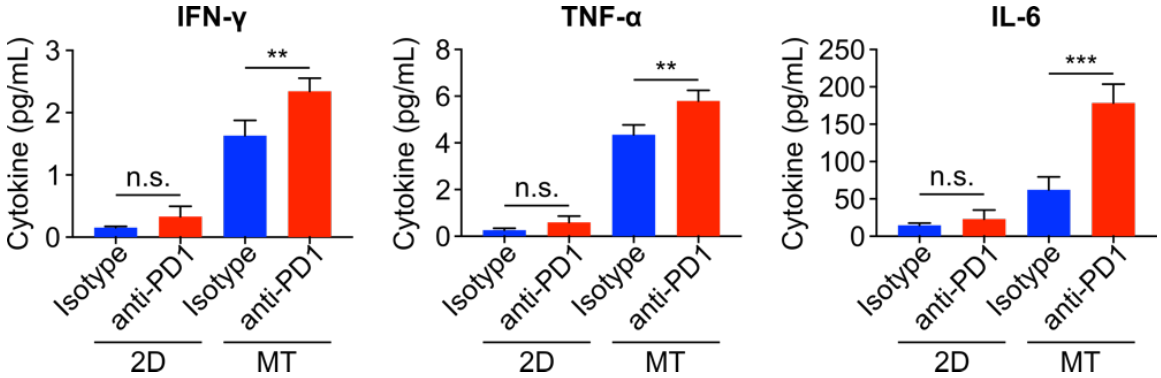

Figure S6. Cytokine analysis of dissociated tumor cells culture ex vivo in 96 well plate (2D) and minitumor chip. Detailed bar graph of cytokine concentrations depicted in Fig. 1G. Cytokine concentrations were analyzed using Biolegend Fireplex assays. Data points from anti-PD1 treated groups were compared with corresponding controls at the same timepoint by student's t-test (n=3, **p<0.01, ***p<0.005).

Table S1. Antibody used in flow cytometry analysis

Antigen	Fluorophore	Host	Vendor	Catalog#	Dilution
CD45	APC	Rat	Biolegend	103111	1:100
CD3	PE/Cy7	Rat	Biolegend	100219	1:200
CD4	Alexa Fluor 700	Rat	Biolegend	100429	1:200
CD8a	APC/Cy7	Rat	Biolegend	100713	1:200
CD19	FITC	Rat	Biolegend	152403	1:100
CD11b	PE	Rat	Biolegend	101207	1:200
F4/80	PerCP/Cy5.5	Rat	Biolegend	123128	1:200
NK1.1	PE/Cy5	Mouse	Biolegend	108715	1:100
Gr-1	PE-eFluor 610	Rat	Invitrogen	61-5931-82	1:100
